# Nutritional State and COPD: Effects on Dyspnoea and Exercise Tolerance

**DOI:** 10.3390/nu15071786

**Published:** 2023-04-06

**Authors:** Angela Tramontano, Paolo Palange

**Affiliations:** 1 Department of Public Health and Infectious Diseases, Sapienza University of Rome, 00185 Rome, Italy; a.tramontano@uniroma1.it; 2 Respiratory and Critical Care, Policlinico Umberto I Hospital, 00161 Rome, Italy

**Keywords:** clinical nutrition, COPD, dyspnoea, exercise tolerance, nutritional state

## Abstract

Chronic Obstructive Pulmonary Disease (COPD) is a disease that is spreading worldwide and is responsible for a huge number of deaths annually. It is characterized by progressive and often irreversible airflow obstruction, with a heterogeneous clinical manifestation based on disease severity. Along with pulmonary impairment, COPD patients display different grades of malnutrition that can be linked to a worsening of respiratory function and to a negative prognosis. Nutritional impairment seems to be related to a reduced exercise tolerance and to dyspnoea becoming a major determinant in patient-perceived quality of life. Many strategies have been proposed to limit the effects of malnutrition on disease progression, but there are still limited data available to determine which of them is the best option to manage COPD patients. The purpose of this review is to highlight the main aspects of COPD-related malnutrition and to underline the importance of poor nutritional state on muscle energetics, exercise tolerance and dyspnoea.

## 1. Introduction

Chronic Obstructive Pulmonary Disease (COPD) is a heterogeneous lung disease characterized by chronic respiratory symptoms such as dyspnoea, cough and sputum production due to abnormalities of the airways (e.g., bronchitis, bronchiolitis) and/or of the alveoli (e.g., emphysema) that cause persistent, often progressive airflow obstruction [1]. Of importance, COPD is associated to high mortality, with around three million deaths annually [2], expected to grow in the next several decades due to the increasing prevalence of smoking and the aging of the population [3,4] and become one of the top three causes of mortality worldwide. Chronic Obstructive Pulmonary Disease emerges as a major cause of chronic morbidity, often in association with other comorbidities including cardiovascular, renal and metabolic diseases. Given these initial considerations, it appears clear that COPD is becoming a significant burden for the public health system that is both preventable and treatable [5]. Recently, the social impact of COPD has also been placed under the spotlight, in that patients affected by this condition tend to limit their activity due to related symptoms such as dyspnoea and poor exercise tolerance. Rehabilitation programs and clinical nutrition share the aim of restoring these lost functions and delaying the grade of impairment linked to the onset of this symptom-related disability.

Even if there is a growing interest in the research field for these topics, clinical awareness of the impact of those aspects is still limited. In this review, we will focus on the mechanisms of nutritional impairment and its consequences on COPD, such as dyspnoea and poor exercise tolerance, with a special reference to poor muscle energetics.

## 2. Nutritional State and COPD

Many COPD patients exhibit a poor nutritional state because of a combination of decreased nutrition from poor micro- and macronutrient intake, systemic inflammatory status and sedentary lifestyle. Disease-related malnutrition can be identified in 30–60% of patients with COPD [6,7,8], and it is usually related to disease severity in terms of airway obstruction [9]. Moreover, abnormal nutritional status and changes in body composition have a substantial negative impact on prognosis, with a higher risk of COPD exacerbations, depression and mortality [10,11,12,13]. The most common pathologies related to malnutrition in COPD patients are cachexia, sarcopenia and osteoporosis.

Cachexia is quite prevalent among COPD patients and can be defined by a 5% weight loss in a year associated with at least three other characteristics such as a reduction in muscle strength and in fat-free mass index (FFMI), fatigue, anorexia and evidence of increased inflammatory markers [14]. The term pulmonary cachexia is addressed to those patients with a severe pulmonary impairment [15]. In fact, many studies have shown that the prevalence of cachexia increases with the worsening of pulmonary function in terms of COPD severity indexes such as the Global Initiative for Chronic Obstructive Pulmonary Disease (GOLD) stage and Medical Research Council (MRC) dyspnoea score [16].

Sarcopenia is also a typical disease of COPD patients and is characterized by low skeletal muscle mass with reduced muscle strength [14]. It has been reported that, while 20–40% of COPD patients have low muscle mass [10,11], 15–21.6% are sarcopenic [16,17]. Of note, the association of malnutrition and sarcopenia (malnutrition-sarcopenia syndrome, MSS) has a stronger impact on mortality than the single condition alone [18].

Osteoporosis can also occur in COPD patients due to anorexia, with consequent reduction in dairy products consumption and reduced daily physical activity. The prevalence of this condition varies from 5 to 60%, based on diagnostic methods, populations studied and disease severity [19]. Vitamin D is fundamental in the regulation of calcium metabolism, with significant association between low serum levels and bone mineral density in COPD patients [20,21]. Moreover, low vitamin D levels have been associated with a reduction in lung function in patients with other pulmonary conditions such as pulmonary fibrosis [22,23]. Vitamin D levels seem to be influenced by serum levels of Vitamin D Binding Protein (VDBP), also known as GC-globulin, encoded by the GC gene; as Vitamin D has a very short life in serum, VDBP stabilizes the molecule and ensures its transport to target tissues. This carrier protein regulates immunological responses such as macrophage activation and neutrophil chemotaxis [24], with an unclear role in lung function decline. Many authors have examined the correlation between VDBP serum levels and polymorphism and COPD [25,26,27].

Considering the well-established positive role of exercise (especially low-impact exercise) on the prevention of osteoporosis [28], it appears clear that reduced exercise tolerance in COPD patients is a major determinant of this condition.

## 3. Leading Causes of Malnutrition in COPD Patients

**Figure 1:**
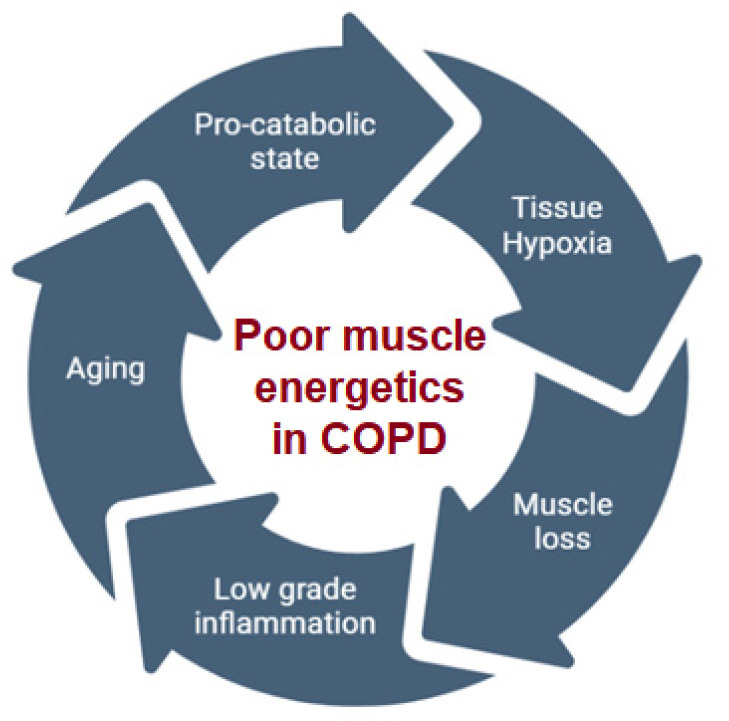
Mechanisms leading to poor muscle energetics.

The principal causes of poor muscle energetics and nutritional impairment in COPD patients are to be found in a disease-induced energy imbalance, due to changes in metabolism, aging, muscle loss and atrophy, tissue hypoxia, a low-grade systemic inflammation and medications [9] (Figure 1).

*Hypermetabolic state.* Many COPD patients are hypermetabolic [29]. Despite this hypermetabolic state, COPD patients tend to reduce their dietary intake [30], due in part to the effects of systemic inflammation that can alter appetite regulation [31], due in part to the intrinsic energetic cost of eating per se. Therefore, COPD patients often tend to limit their dietary intake to reduce disease-related symptoms (dyspnoea) [32]. Moreover, while in normal subjects the physiological response to a condition of semi-starvation is a reduction of metabolic rate and in body-protein turnover, COPD patients may have an elevated energy expenditure even at rest, with an increased protein turnover [33].

*Muscle wasting.* This pro-catabolic state is one of the principal determinants of muscle wasting. The progressive muscle mass loss is also determined by a change in oxidative metabolism in peripheral muscle, with a major susceptibility to oxidative stress and less energy efficiency if compared to normal subjects. The analysis of skeletal muscle fibres from COPD patients shows a shift from type I to type II fibres [34,35,36,37] and a reduction of their oxidative capacity [38]. Moreover, type II fibres seem to be more vulnerable to inflammation and hypoxia-mediated atrophy [34]. This decline in the oxidative mechanisms of compensation may speed up muscle mass depletion, linking muscle quality to muscle quantity [39]. These aspects have proven to have a negative effect on exercise tolerance [40]. In fact, the above-mentioned structural and metabolic abnormalities translate into a reduction of muscle strength and endurance, especially in the lower limbs [41,42,43,44]. The effects of cachexia do not spare respiratory muscles: in fact, the diaphragm muscle mass, length, thickness and strength of COPD patients are reduced if compared to non-cachectic patients [45]. Furthermore, in COPD patients without body weight abnormalities, variations in diaphragm characteristics seem to follow those of normal individuals. Thus, malnutrition can be easily associated with progressive diaphragmatic weakness, and the effect is more pronounced in those patients with advanced lung disease.

*Hypoxia and hypercapnia.* In COPD patients, hypoxia and hypercapnia can also negatively affect muscle function [46]. Chronic hypoxemia [47] and the often-coexistent anaemia [48] can lead to muscle hypoxia due to a reduction in oxygen delivery that may lead to systemic inflammation, elevated protein turnover and deficient muscle regeneration [49,50,51]. All these mechanisms are responsible for decreasing muscle mass and reducing muscle oxidative capacity [52], leading to impaired muscle endurance. It is also known that in normal individuals hypercapnia can induce muscle dysfunction [53], and that acidosis may alter protein homeostasis [54]. Recent findings have shown the negative effects of hypercapnia on protein synthesis and fibre atrophy in COPD patients [55].

*Aging.* Aging is associated with variation in body composition with the loss of free-fat mass, especially muscle tissue, associated to a progressive increase in fat stores. Muscle mass depletion leads to reduced muscle strength and decreased basal metabolic rate; this results in a reduction in exercise tolerance. Aging also affects exercise tolerance by other means. For instance, osteoporosis, which is more common in the elderly, can cause vertebral fractures leading to spinal abnormality, decreased diaphragmatic excursion and the impairment of respiratory secretion clearance.

*Comorbidities.* In COPD, comorbidities such as chronic heart failure (CHF) and chronic kidney disease (CKD) have a fundamental role in the resulting muscle wasting syndrome. In a recent review, Dubè and Laveneziana highlighted how many similarities can be found in muscle loss between CKD, CHF and COPD, considering that all these conditions are characterized by a low-grade systemic inflammation, reduction in daily activities and similar muscle structure changes such as a rearrangement from type I to type II fibre distribution [56].

*Inflammation.* In COPD patients, low-grade systemic inflammation may determine the activation of different cellular pathways that can lead to muscle atrophy and muscle dysfunction. Reid et al. demonstrated that Tumour Necrosis Factor-alpha (TNF-α) and other cytokines can directly inhibit muscle contraction [57]. Historically, the “spill over” theory suggested that systemic inflammation derives primarily from the lungs and later it spreads to the whole body through the bloodstream [58]. However, recent findings have shown that extrapulmonary involvement in COPD may begin simultaneously with lung disease, as a linear effect of the same damage. This hypothesis is supported by the lack of an association between pulmonary and serum or other organ inflammatory marker levels, and by the fact that muscle changes often precede the onset of pulmonary abnormalities. In patients with COPD, different articles showed an increase in white blood cells and in different biomarker levels, such as C-reactive protein and other pro-inflammatory cytokines [59,60].

*Drugs.* Different drugs are utilized in COPD that can determine alterations in skeletal muscles. Corticosteroids, especially in their systemic formulation, can mediate both acute and chronic myopathies. Corticosteroids are mainly utilized for acute exacerbations of COPD and in patients with very advanced disease. Chronic exposure to even moderate doses of corticosteroids, however, should be avoided because it can induce a chronic myopathy characterized by type II fibre atrophy, changes in carbohydrate metabolism and a negative balance in protein metabolism [61]. All these alterations result in muscle weakness mainly affecting proximal muscle groups. Anticholinergic drugs, at standard doses, seem not to have relevant effects on skeletal muscle function; high dosages, however, can determine a reduction in contractile reaction time [62]. Finally, a wide range of drugs utilised in cardiovascular diseases, often in comorbidity with COPD, may have noxious effects on skeletal muscles: β-blockers may promote muscle fatigue [63], calcium channel blockers can diminish contraction and decrease muscle regeneration [64], statins may determine a specific myopathy [65] and some diuretics may lead to an alteration in blood ion levels, potentially tampering with muscle function [66].

## 4. Nutritional Assessment

With the aim of an early identification of malnutrition, COPD patients should be screened during routine visits or every 6–12 months for pulmonary cachexia or alarming weight loss. Serial measurements of body weight offer the simplest screen for nutritional assessment. In patients with weight loss >5%, a weight <90% of ideal body weight (IBW), or a body mass index (BMI) ≤20 kg/m^2^ pulmonary cachexia should be suspected. Of note, BMI accuracy is limited, not considering age or sex, and fails to distinguish between proportions of bone, lean body mass, or fat mass. However, concerning COPD patients, low BMI has been correlated to a reduced median survival [2]. A more accurate index to evaluate body composition is the Fat Free Mass Index (FFMI), obtained by dividing Fat-Free Mass (FFM, measured via bioelectrical impedance) to height in square meters (FFMI = FFM/height^2^). Low FFMI is defined as FFMI <15 kg/m^2^ in women and FFMI <16 kg/m^2^ in men [67]. Bioelectrical impedance analysis (BIA) is a convenient, portable and low-cost measure of weight, and FFMI seems to be more sensitive in terms of prognostic accuracy if compared to BMI; therefore, it should be used for the routine clinical evaluation of COPD patients [68,69].

The presence of sarcopenia can be evaluated using Dual-energy X-ray Absorptiometry (DXA), which can differentiate fat, lean body mass and bone tissue [70]. Even if considered the gold standard to assess body composition, it is used mainly in the research setting but, because of is extremely low radiation dose, should be preferred to Computed Tomography (CT) [71]. Magnetic Resonance Imaging (MRI) can also help in determining body composition, but the excessive cost limits use to the research setting.

There is increasing interest in the use of ultrasonography to assess nutritional status. The main fields of application by now are the evaluation of diaphragm thickness [72] and the echo intensity of the rectus femoris [73].

Various screening tests have been developed to assess nutritional risk, including the Patient-Generated Subjective Global Assessment (PG-SGA) [74] and the Mini Nutritional Assessment (MNA) [75,76]. Other tools include the Nutritional Risk Screening tool 2002 (NRS-2002) [77,78] and the most recent Global Leadership Initiative on Malnutrition (GLIM) to assess malnutrition and the European Working Group on Sarcopenia in Older People 2 (EWGSOP2) criteria to determine sarcopenia risk and severity [79].

## 5. Effects of Poor Nutritional State on Exercise Tolerance and Dyspnoea

In COPD, the factors dictating exercise tolerance are limited ventilatory response, dynamic hyperinflation and dyspnoea.

The major determinants of a proper ventilatory response to exercise (V′_E_) are V′_CO2_ (the metabolic component) and PaCO_2_ (the control “set point”); ventilatory efficiency (V′_E_/V′_CO2_) is defined as the amount of ventilation necessary to remove a certain amount of CO_2_ and regulates ventilatory adaptation to exercise. Chronic Obstructive Pulmonary Disease patients are usually unable to increase alveolar ventilation in response to increasing V′_CO2_ due to mechanical constraints from dynamic hyperinflation during exercise and altered ventilation/perfusion relationships [80].

The dead space fraction (V_D_/V_T_) and the limit to which the ventilatory system is stressed can also affect ventilatory response to exercise. Because of respiratory flow limitation that could be present even at low–moderate levels of exercise, patients with COPD need to increase end-expiratory lung volumes and use their inspiratory reserve volume to raise V′_E_ as a response to increased metabolic activity. Subsequent hyperinflation determines the flattening of the diaphragm and decreases its contractile strength, determining increased muscle fatigue and increased dyspnoea at any given level of ventilation. It has also been shown that dyspnoea intensity rises as V′E increases [81].

Ventilatory limitation seems to mostly affect the walking capacity rather than cycling capacity of COPD patients: in fact, performing shuttle walking tests, they exhibit a more pronounced V′_E_/V′_CO2_ response compared to cycling; this seems to be mainly associated with a decreased lung gas exchange efficiency but also to a higher neurogenic afferent stimulus to the respiratory centres from vagal pulmonary receptors and hemodynamic adaptation [82]. These aspects are relevant because the data derived from COPD patients during cycling may not properly indicate their ventilatory and metabolic needs for daily activities such as walking.

Skeletal muscle hypoperfusion and deconditioning have a significant role in exercise tolerance and can bring to early onset lactic acidosis and higher V′_CO2_, and therefore ventilatory demand, for specific exercise load [83]. In severe COPD patients, exercise tolerance may be constrained by limb muscle fatigue rather than ventilatory limitation [84]. As mentioned before, many COPD patients present various grades of muscular impairment, from mild deconditioning to severe sarcopenia, correlating to disease severity.

Compared with non-depleted patients, malnourished COPD patients seem to produce a blunted ventilatory response at maximal exercise, maybe due to more dynamic hyperinflation [85]. A recent study by Teopompi et al. re-evaluated these hypotheses and showed that FFM depletion is crucial in reducing exercise capacity, irrespective of ventilatory limitation in COPD patients [86]. Moreover, FFM depletion was associated with a limited cardiovascular response to exercise and leg fatigue, but not to exertional dyspnoea.

Moreover, impaired oxygen transport can be identified as one of the major reasons for reduced exercise tolerance in COPD patients. Shan et al. demonstrated that the peak V_O2_ and peak O_2_ pulses of male COPD patients with nutritional risk were significantly lower than those with no nutritional risk, with a negative correlation with NRS-2002 score [87]. These results show that this lower oxygen uptake in COPD patients with nutritional risk could be determined by an impairment in oxygen transport affecting oxygen utilization and subsequently exercise capacity for a reduced muscle oxygen delivery [88].

## 6. Strategies to Improve Nutritional State and Exercise Tolerance

Muscle impairment and poor nutritional state in COPD tend to coexist and can cooperate in worsening exercise tolerance and dyspnoea. Nutritional intervention may be useful to prevent muscle wasting and exercise impairment to achieve a better quality of life for COPD patients.

The rationale for nutritional intervention is clear and recommended by the latest GOLD report [1]; a combination of exercise, the control of inflammation and nutritional support may be used to prevent all the negative effects of the development of pulmonary cachexia. Supplemental nutrition alone is usually inadequate to boost functional exercise capacity. Increasing the intake amount of specific macronutrients can be challenging in patients with advanced lung disease because of disease-related symptoms: for example, dyspnoea can interfere with food preparation and consumption, while chronic sputum production may alter the taste of food; moreover, the flattening of the diaphragm may determine early satiety. A reduction in breathing function could theoretically limit caloric expenditure as improving lung function could reduce dyspnoea, therefore improving macronutrient intake, and empower adherence to rehabilitation programs [89].

Exercise is still the only known intervention strategy to reverse some of the skeletal muscle abnormalities typical of COPD patients. In fact, it has come to light that exercise, in the form of pulmonary rehabilitation, is the most effective non-pharmacological intervention in improving exercise capacity and dyspnoea [90]. Moreover, in comorbid patients where cardiovascular, metabolic and respiratory disorders coexist there is a growing interest in considering exercise as the “real polypill” in the emerging scenario of regenerative medicine [91]. There is still a strong debate on which mode of exercise could be most effective in improving the long-term outcomes in COPD patients. It appears clear that resistance training can reduce sarcopenia and promote muscle fibre hypertrophy [92]. High-intensity exercise could also be beneficial for COPD patients, but in patients with severe disease dyspnoea sensation may limit the amount of sufficiently extended periods of high intensity training [93]. Another approach more suitable for this group of patients may be interval training, with short periods of high-intensity exercise. Strategies to reduce dyspnoea sensation in these rehabilitation programs can include supplemental oxygen therapy [94] or Heliox [95]. Recent findings suggest that inspiratory muscle training can reduce dyspnoea sensation by augmenting inspiratory muscle strength and endurance [96]. However, the effects of respiratory muscle training are still unclear, with further investigation needed [97]. Despite all the benefits provided by exercise, the structural abnormalities in limb muscle are not fully reversible by exercise alone and the improvement obtained with rehabilitation programs tends to decline to the baseline in the following 12–18 months. Hence, there is a strong interest in possible pharmacological treatments associated with exercise-based training.

Since systemic inflammation and oxidative stress have been postulated to be aetiological factors of muscle dysfunction in COPD, and given the role that nutritional antioxidants such as vitamin C and retinols may have in preventing lung tissue damage by proteases and in protecting the body against the development of the disease, a possible strategy could be to utilize antioxidant supplementation. A small study on nine COPD patients showed that antioxidant therapy with N-acetylcysteine (NAC) determined an increase of 25% in quadriceps endurance when compared to a placebo [98]. A recent study showed that supplementation with NAC and ascorbate improved the nutritional status and oxidative status of COPD patients [99]. In contrast, a recent work by Hureau et al. [100] showed that although vitamin C promoted indicators for antioxidant capacity, reduced inflammatory markers and improved neuromuscular fatigue resistance it failed to ameliorate dyspnoea on exertion and cycling exercise tolerance in COPD patients. Tocotrienols have the ability to modulate the progression of COPD by targeting inflammatory pathways, making them potential candidates for novel therapeutic approaches [101]. Polyunsaturated fatty acids (PUFA) mediate various inflammatory and metabolic pathways, which may be crucial in the pathogenesis of muscle dysfunction in COPD: their beneficial effects on exercise capacity have already been demonstrated [102]. A recent meta-analysis showed that there is a positive effect of omega-3 PUFA supplementation on overall body muscle mass and strength [103]. A more recent study by Fekete et al. examined their effects on COPD patients and found that their supplementation could be positively associated with nutritional status, inflammatory parameters, respiratory medication intake and exacerbation rates [104]. However, this evidence is still preliminary, with the necessity of further studies.

Many studies on vitamin D supplementation in patients with pulmonary disease showed its beneficial effects but have not proven a clear benefit [105]. A study by Sundar et al. showed that in vitamin D receptor knockout mice there is an earlier development of emphysema because of an increased production of matrix metalloproteinases, with an imbalance in protease/antiprotease expression [106]. Evidence from clinical trials shows that vitamin D supplementation can decrease COPD exacerbation. A double-blind placebo control randomized clinical trial has shown that the oral administration of vitamin D decreases COPD exacerbation and improves pulmonary function in terms of FEV1 (forced expiratory volume-1) [107]. Another study by Sluyter et al. also shows that the supplementation of a long-term monthly high dose of vitamin D can improve lung function in vitamin D-deficient COPD patients [108].

A targeted medical nutrition program could also help COPD patients with a multimodal approach. Calder et al. showed that targeted medical nutrition with high-dose omega-3 fatty acids, vitamin D and high-biological-value protein is well tolerated with a good safety profile, with positive effects on exercise-induced fatigue and dyspnoea. Therefore, such kinds of intervention could be beneficial in the nutritional and metabolic support of COPD pre-cachectic and cachectic patients [109].

It is important to underline that not every macronutrient has a potentially positive effect on the prevention of nutritional impairment in COPD patients, with some of them hiding deleterious effects on respiratory responses and exercise tolerance. Among potential harmful foods, a negative association between the consumption of processed and red meats and pulmonary function has been described. Even if a high-protein diet could potentially restore muscle strength in COPD patients, processed red meat should be avoided since it is rich in nitrites, which can generate reactive nitrogen species (e.g., peroxynitrite) with subsequent nitrosative stress that can amplify lung inflammatory processes. Of note, in animal models a chronic exposure to nitrite has been associated with emphysema-like lung damage [110]. Moreover, meat contains high saturated fatty acid (SFAs) levels that can stimulate systemic inflammation [111]. By contrast, an increased intake of low-fat dairy products [112] as well as of short- and medium-chain SFAs may have protective effects on lung function, possibly through an anti-inflammatory action [113].

Another aspect to highlight is the potentially deleterious effect of a high-carbohydrate diet on COPD patients. Hyperglycaemia may enhance oxidative stress-related inflammatory responses [114], in part through the formation of advanced glycation end-products (AGEs) [115], and has been associated with impaired lung function in COPD patients [116]. Moreover, an excess carbohydrate supply may lead to a worsening in pulmonary function, due to the activation of lipogenesis pathways, with an excess production of carbon dioxide and subsequent increase in respiratory rate, thus dyspnoea. It has been proposed that increasing the caloric intake of COPD patients through a high-fat diet may be more helpful due to the reduced production of the metabolic carbon dioxide of fat. Kuo et al. demonstrated that in clinically stable ambulatory COPD patients a high-fat diet is more beneficial than high-carbohydrate diet, with lower levels of carbon dioxide production, oxygen consumption and minute ventilation. [117]. However, some other studies appear to provide contradictory evidence regarding this aspect [118,119,120]; thus, further research is needed to determine what type of calorie supplementation is the best for COPD patients.

## 7. Conclusions

In COPD, poor nutritional state is a determining factor in developing muscle weakness, exercise intolerance and ultimately dyspnoea. An individualized nutritional therapy should be instituted as soon as possible with every COPD patient to prevent nutritional impairment and to support immune function, muscle strength and exercise tolerance. With the aim of a multimodal approach to the disease and for a better understanding of the mechanisms underlying nutritional impairment, it is necessary to enrich the already florid literature on this topic. Future studies are needed to provide more information on the role of nutritional status in combination with aging and levels of daily activity in disease progression with the aim of assuring a better quality of life for COPD patients [121].

## Data Availability

Not applicable.
